# Pharmacokinetics of immediate and sustained-release formulations of paroxetine: Population pharmacokinetic approach to guide paroxetine personalized therapy in chinese psychotic patients

**DOI:** 10.3389/fphar.2022.966622

**Published:** 2022-09-12

**Authors:** Xiao-lin Li, Shan-qing Huang, Tao Xiao, Xi-pei Wang, Wan Kong, Shu-jing Liu, Zi Zhang, Ye Yang, Shan-shan Huang, Xiao-jia Ni, Hao-yang Lu, Ming Zhang, Yu-guan Wen, De-wei Shang

**Affiliations:** ^1^ Department of Pharmacy, The Affiliated Brain Hospital of Guangzhou Medical University, Guangzhou, China; ^2^ Medical Research Center, Guangdong Province People’s Hospital, Guangdong Academy of Medical Sciences, Cardiovascular Institute, Guangzhou, China; ^3^ Guangdong Engineering Technology Research Center for Translational Medicine of Mental Disorders, Guangzhou, China

**Keywords:** paroxetine, Chinese, dose-dependent, formulation, population pharmacokinetics

## Abstract

Paroxetine is one of the most potent selective serotonin reuptake inhibitors (SSRIs) approved for treating depression, panic disorder, and obsessive-compulsive disorder. There is evidence linking genetic polymorphisms and nonlinear metabolism to the Paroxetine’s pharmacokinetic (PK) variability. The purpose of the present study was to develop a population PK (PPK) model of paroxetine in Chinese patients, which was used to define the paroxetine’s PK parameters and quantify the effect of clinical and baseline demographic factors on these PK characteristics. The study included 184 inpatients with psychosis (103 females and 81 males), with a total of 372 serum concentrations of paroxetine for PPK analyses. The total daily dosage ranged from 20 to 75 mg. One compartment model could fit the PKs characterize of paroxetine. Covariate analysis revealed that dose, formulation, and sex had a significant effect on the PK parameters of paroxetine; however, there was no evident genetic influence of *CYP2D6* enzymes on paroxetine concentrations in Chinese patients. The study determined that the population’s apparent distribution volume (V/F) and apparent clearance (CL/F), respectively, were 8850 and 21.2 L/h. The CL/F decreased 1-2-fold for each 10 mg dose increase, whereas the different formulations caused a decrease in V/F of 66.6%. Sex was found to affect bioavailability (F), which decreased F by 47.5%. Females had higher F values than males. This PPK model described data from patients with psychosis who received paroxetine immediate-release tablets (IR-T) and/or sustained-release tablets (SR-T). Paroxetine trough concentrations and relative bioavailability were different between formulations and sex. The altered serum concentrations of paroxetine resulting from individual variants and additive effects need to be considered, to optimize the dosage regimen for individual patients.

## 1 Introduction

One of the most potent selective serotonin reuptake inhibitors (SSRIs), paroxetine is frequently prescribed to treat depression, anxiety, panic disorder, and obsessive-compulsive disorder (Venkatakrishnan and Obach, 2005; Nishimura et al., 2016). The recommended daily dose of paroxetine is generally 20 mg, which is gradually increased by 10 mg/day (once daily) every week until a maximally tolerated dose is reached (Nishimura et al., 2016). The guidelines from the Arbeitsgemeinschaft für Neuropsychopharmakologie and Pharmakopsychiatri (AGNP) list the therapeutic range as 20–65 ng/ml (Hiemke et al., 2018). After oral treatment, paroxetine is mostly absorbed by the digestive system and partly metabolized by *CYP2D6* into inactive metabolites. Paroxetine easily accumulates *in vivo* after repeated administration, and the pharmacokinetic (PK) characteristics alter with dosage, leading to nonlinear PKs and increased exposure with time (Bourin et al., 2001). For healthy subjects, the overall average half-life of elimination is approximately 21 h (Nishimura et al., 2016). For different formulations, immediate-release paroxetine is rapidly absorbed compared with sustained-release tablets (T_max_ 5.2 versus 8 h) (Foster and Goa, 1997; DeVane, 2003; U.S. Food and Drug Administration, 2014; U.S. Food and Drug Administration, 2015). A vast volume of distribution reveals that paroxetine is extensively distributed in the body and is highly bound to plasma proteins (approximately 95%) (U.S. Food and Drug Administration, 2015). It is well acknowledged that genetic polymorphisms and nonlinear metabolism are linked to paroxetine’s PK variability. To determine the paroxetine dose, therapeutic drug monitoring (TDM) and the psychiatrist’s experience are typically applied.

Previous PK models have assumed that paroxetine is eliminated by at least two different kinetic mechanisms, including a low-affinity linear process and a high-affinity saturable one. Human paroxetine metabolism is most likely dominated by *CYP2D6* (high affinity) and *CYP3A4* (low affinity) (Sindrup et al., 1992; Carvalho Henriques et al., 2020). In some individuals, *CYP1A2* may also be important for paroxetine metabolism (Jeppesen et al., 1996; Jornil et al., 2010; Lin et al., 2010). Some studies have suggested that serotonin (5-HT) transporter (SERT) candidate genes (such as *SLC6A4*) and serotonin receptor genes (such as *HTRLPR*) may influence paroxetine treatment response (Wilkie et al., 2009; Ishiguro et al., 2011; Marazziti et al., 2020). The *CYP2D6* genetic polymorphism is widely documented as a primary predictor of paroxetine PK variability; thus, most PK studies of paroxetine have focused on the prediction of the paroxetine concentrations and *CYP2D6* genotype (Feng et al., 2006; Findling et al., 2006; Mikami et al., 2013; Nishimura et al., 2016). In clinical practice, the purpose of genotyping is to correlate genotypes to individual therapy responses and to alter treatment dosages accordingly. A previous study of Korean subjects examined the influence of sex, age, body weight, diagnosis, and serum albumin level on paroxetine plasma concentrations, with age and daily dose identified as the major variables (Kim et al., 2015). Another study of older people evaluated the influence of parameters such as race, sex, age, weight, and *CYP2D6* genotypes on paroxetine PKs and discovered that weight and *CYP2D6* polymorphisms had an impact on maximum velocity (Vm), whereas sex had an impact on the volume of distribution (Feng et al., 2006). Furthermore, age, weight, sex, *CYP2D6* genotypes, and daily dosage were included as variables in a population PK (PPK) analysis of Japanese patients, and sex and genotypes were found to be significant (Nishimura et al., 2016).

With changes in pharmacodynamics and PKs, more concurrent ailments, and the increased combination of differential situations, the variables that impact PKs in the paroxetine population are complicated, with apparent individual variances. A study showed that the concentration of paroxetine showed an exponential linear curve with increasing doses, especially at lower doses, and the variance was not reflected at high doses (Carvalho Henriques et al., 2020). Most patients experienced an unequal rise in their plasma drug levels with increasing doses. It is possible that this was due to the saturation of enzyme and its self-inhibition (Sawamura et al., 2004). Given that trough concentration is closely connected with clinical outcomes, paroxetine trough concentrations are examined to help with dose modification (Kim et al., 2015). Additionally, the relationship between paroxetine trough concentrations and area under the curve (AUC) is similar between sustained- and immediate-release paroxetine (U.S. Food and Drug Administration, 2014; U.S. Food and Drug Administration, 2015); therefore, both formulations can be monitored using the same TDM approach.

Numerous pieces of research have characterized the PKs of paroxetine with immediate-release; even so, few PPK studies have characterized the PKs of formulations with both immediate- and sustained-release. Although some paroxetine PPK studies have been established to characterize factors for paroxetine, they have not been fully characterized in the Chinese population. In addition to identifying the baseline demographic and covariate characteristics that have a substantial impact on paroxetine PKs, this modeling study was conducted to describe the PPKs of immediate- and sustained-release paroxetine at different dosages.

## 2 Methods and materials

### 2.1 Subjects and study protocol

We conducted a retrospective analysis using TDM data of psychiatric inpatient treatment on paroxetine between 1 January 2019 and 31 May 2021 in the Affiliated Brain Hospital of Guangzhou Medical University (China). The Medical Ethics Committee of the Affiliated Brain Hospital of Guangzhou Medical University gave the study approval, and it was conducted in accordance with the Declaration of Helsinki. Paroxetine tablets (including immediate- and sustained-release) were administered using different daily doses (doses ranging from 20 to 75 mg/day) that were constantly adjusted throughout the treatment period.

Patients were eligible if they fulfilled the following criteria: 1) took paroxetine with monitoring of serum drug concentration, 2) one or more serum concentration data could be acquired at various dosages, and 3) the patients’ information was available in medical records. Exclusion criteria were as follows: 1) by evaluating if paroxetine concentrations were zero or less than the lower quantification limit or more than the upper limit, patients were suspected of temporary noncompliance, 2) serum samples were obtained at inappropriate times, or the researchers determined the patient was unsuitable for inclusion.

The advantage of population pharmacokinetics is that pharmacokinetic parameters may be determined using sparse blood collection data, and there is no need to collect steady-state blood concentration data. Finally, we gathered a range of information, including demographic characteristics (age, sex, height, body weight, BMI), drinking or smoking status, serum concentration, and concomitant medication (such as aripiprazole, tandospirone, risperidone, metoprolol). Biochemical parameters, such as liver function (alanine transaminase, aspartate aminotransferase, serum albumin), renal function (serum creatinine, blood urea nitrogen), blood lipids (total cholesterol), and the plasticity of prolactin, were obtained from patient records.

### 2.2 Analytical procedures

Shimadzu 20A HPLC system, which includes an autosampler, a degassing unit, two LC-20AD pumps, and a column oven, was used to measure the serum concentration of paroxetine (Shimadzu Corporation, Kyoto, Japan). A Shimadzu LCMS-8040 (Shimadzu Corporation) set with an electrospray ionization source (ESI) working in positive mode was used for MS detection in multiple-reaction monitoring mode (MRM). Paroxetine and paroxetine-d4 isotope (Toronto, Canada) were used. Blood samples were obtained from the patient’s veins using procoagulant tubes. Then, serum was collected by centrifugation at high speed for 3 minutes, and finally, samples were stored in a −80°C refrigerator. The acetonitrile protein precipitation method was employed to extract analytes from 100 μL serum using 500 μL acetonitrile.

At 35°C, the separation was performed on an Agilent Eclipse XDB C18 column (4.6 mm × 50 mm, 1.8 μm). The ion pairs employed for quantitative analysis were m/z 330.05→m/z 191.8 (paroxetine), m/z 334.05→m/z 195.8 (paroxetine-d4) with a full running duration of 90 s for each sample. The ratio of A to B in the mobile phase was 1:1 with the flow rate being 0.5 ml/min. A was a mixture of methanol-water containing 5 mmol/L ammonium formate (75:25, v/v), while B was pure methanol. The calibration curve was 5, 10, 100, 200, 300, 400, and 500 ng/ml. The linear assay covered the range of 5–500 ng/ml (*R* ^2^ > 0.99). The stability was good, and both the intra-day and inter-day precisions’ relative standard deviations (RSD%) were within 15%.

### 2.3 Population PK modeling

#### 2.3.1 Model development

The PPK analysis was performed using nonlinear mixed-effects modeling (NONMEM^®^, version 7.3), originating from Icon Development Solutions, United States A one-compartment (subroutine ADVAN 2) with the first-order conditional estimation with interaction (FOCE-I) was assumed to estimate model parameters. Pirana software (version 2.9.0, Uppsala University, Sweden) was used to model design and validation. Using an R script (version 4.1.2), the diagnostic charts of NONMEM^®^ results were tabulated and summarized graphically (Huang et al., 2021).

There were few concentration samples to describe the PKs of paroxetine in the study. Data on concentration were collected 10–22.5 h after the most recent dosage. The trough concentration was collected during the elimination phase. Even if there were errors in the serum sampling time, the trough concentrations’ changes were not significant (Xiao et al., 2021). The absorption rate constant (Ka) was set to previously published values of 0.908/h due to the absence of serum samples throughout the absorption phase (Venkatakrishnan and Obach, 2005; Nishimura et al., 2016). Similarly, absorption lag time could not be assessed. Therefore, a one-compartment PK model was evaluated as the structural model. The following are the exponential random-effects models that were used to characterize the interindividual variability (IIV) and residual error variability models (Eq. 1).

$$P_{i}=P_{tv}\times e^{\eta i}$$ (1)

Where P_i_ is the *i*th individual’s PK parameter value, P_tv_ is the parameter’s population typical value. The distinction between P_i_ and P_tv_, which assumed a normally distributed with a variance of ω2 and a zero mean, is represented by ηi.

#### 2.3.2 Covariate testing

A covariate model was developed using the equations with the forwarding inclusion and backward elimination method. Each covariate was added to the basic model step by step during the forward inclusion phase.

The continuous covariates were evaluated using a linear Eq. 2 or Eq. 3: age, body weight, height, BMI, daily dose, serum paroxetine concentration, aspartate aminotransferase, aspartate aminotransferase isoenzyme, alanine transaminase, total protein, serum albumin, total bilirubin, direct bilirubin, serum creatinine, blood urea nitrogen, total cholesterol, and the plasticity of prolactin.

$$P_{i}=P_{tv}\times {\;e}^{\eta i}\times \left(1+{\;\theta }_{COV}*\left(COV-{COV}_{mid}\right)\right)$$ (2)

Or

$$P_{i}=P_{tv}\times {\;e}^{\eta i}\times {\left(COV/{COV}_{mid}\right)}^{\theta cov}\;)$$ (3)

where COV represents the continuous covariate, COV_mid_ represents the middle of the corresponding covariate. θcov is a factor used to adjust the *i*th PK parameter.

In addition, the categorical variables were investigated with the linear model in Eq. 4, including sex, formulation, and combination medication. The following equation shows the effect of the categorical covariate (Eq. 5).

$$P_{i}=P_{tv}\times e^{\eta i}\times \left(1+{\;\theta }_{COV}\times COV\right)$$ (4)

Or

$$P_{i}=P_{tv}\times {e\;}^{\eta i}\times \theta_{COV}$$ (5)

Where, COV for the sex covariate is 1 (representing female) and 0 (representing male). If the patient is given concomitant drugs, the combination medication covariate is 1, otherwise 0. The formulation was assigned a categorical variable (COV = 1 for immediate-release tablet; COV = 0 for sustained release tablet).

Also, if the variant influences both CL and V. Considering the following equation shows the effect of covariates (Eq. 6)

$$F1=1+\theta_{COV}\times COV$$ (6)

During the forward inclusion process, when the inclusion of a covariate resulted in a reduction of the objective function value (OFV) > 6.63 (*p* < 0.01, degree of freedom = 1), it was considered statistically meaningful, and they should be retained in the base model. Once no additional covariates could be added, the full model was constructed. During the covariates were gradually removed from the full model, the covariates were deemed as significant for the model when their removal increased the OFV >10.83 (*p* < 0.001, degree of freedom = 1). When no further covariates could be removed, the final model held.

#### 2.3.3 Model evaluation

To evaluate the PK parameter estimates of the final PPK model, the stability of models was validated by using OFV value, relative standard error (RSE), goodness-of-fit plots (GOF), and the normalized prediction distribution error (NPDE). Models created from unordered data can be evaluated using the NPDE plots as an external or internal evaluation. Based on nonparametric bootstrapping without stratification (*n* = 1,000), the NPDE was determined for each dataset using the R program and an associated package (version 2.0). The NPDE diagrams were also created using R software (version 4.1.2).

#### 2.3.4 Model simulations

##### 2.3.4.1 Relationship of paroxetine concentration with covariates identified

In the model-based simulations, typical parameter estimates were used to simulate various populations. To assess the changes occurring with oral administration of immediate-release paroxetine at 20, 30, 40, 50, and 60 mg once daily for 100 days, we simulated the dose course in males and females based on population typical values.

Additionally, 1,000 patients’ paroxetine trough concentrations (24 h post-dose) for immediate-release and sustained-release paroxetine (25 mg/50 mg, once daily for 100 days) were simulated to compare the variation of immediate- and sustained-release paroxetine. Individual predicted (IPRED) data were employed to make the analysis. 1000 IPRED could obtained by 1,000 simulations for a given dosage. 95% confidence interval patients were resampled from the individual predicted data to develop a credible combination of multivariable. Box plots were used to summarize the influence of each covariate.

The purpose of these simulations was to explore the effects of cumulated dose, formulation, and sex on paroxetine serum concentrations in different clinical populations.

## 3 Results

### 3.1 Data set characteristics

Overall, a total of 184 subjects with psychosis (103 females, 81 males), and a total of 372 serum concentrations of paroxetine for PPK analyses were included in the study. The baseline characteristics of enrolled patients (such as age, sex, concomitant medications, and liver function) are summarized in Table 1.

**TABLE 1:** Demographic characteristics and clinical data in this study (*n* = 184).

Characteristic	N (%)	Median (range)
Number of subjects	184	
Serum of paroxetine concentration data (ng/ml)	372	60.55 (2.62–447.87)
Age (years)		37.5 (15–90)
Body weight (kg)		60 (58–96)
Height (cm)		164.5 (142–180)
Sex		
Male	81 (44%)	
Female	103 (56%)	
Concomitant medications		
Metoprolol	10 (5.4%)	
Olanzapine	31 (16.8%)	
Risperidone	24 (13.0%)	
Tandospirone	26 (14.1%)	
Liver function		
aspartate aminotransferase (AST, U/L)		17 (9–77)
alanine transaminase (ALT, U/L)		16 (5–100)
total protein (TP, g/L)		67.4 (48.3–105.4)
serum albumin (ALB, g/L)		41.6 (27.8–55.3)
total bilirubin (TBIL, μmol/L)		11.1 (3.2–47.2)
direct bilirubin (DBIL, μmol/L)		3.3 (1–14.2)
Renal function		
serum creatinine (SCr, μmol/L)		65 (34–120)
blood urea nitrogen (BUN, mmol/L)		4.03 (1.32–8.59)
Glucolipid metabolism		
total cholesterol (TC, mmol/L)		4.68 (2.43–105.4)
the plasticity of prolactin (PRL)		496.99 (61.69–5,692.14)
Paroxetine formulation related to concentrations n = 372		
tablet	318 (85.5%)	
sustained-release tablets	54 (14.5%)	

Notes: Continuous variables are presented as median (range) and categorical variables are presented are presented as frequency (percentage).

### 3.2 Model development

#### 3.2.1 Population pharmacokinetic model

**FIGURE 1:**
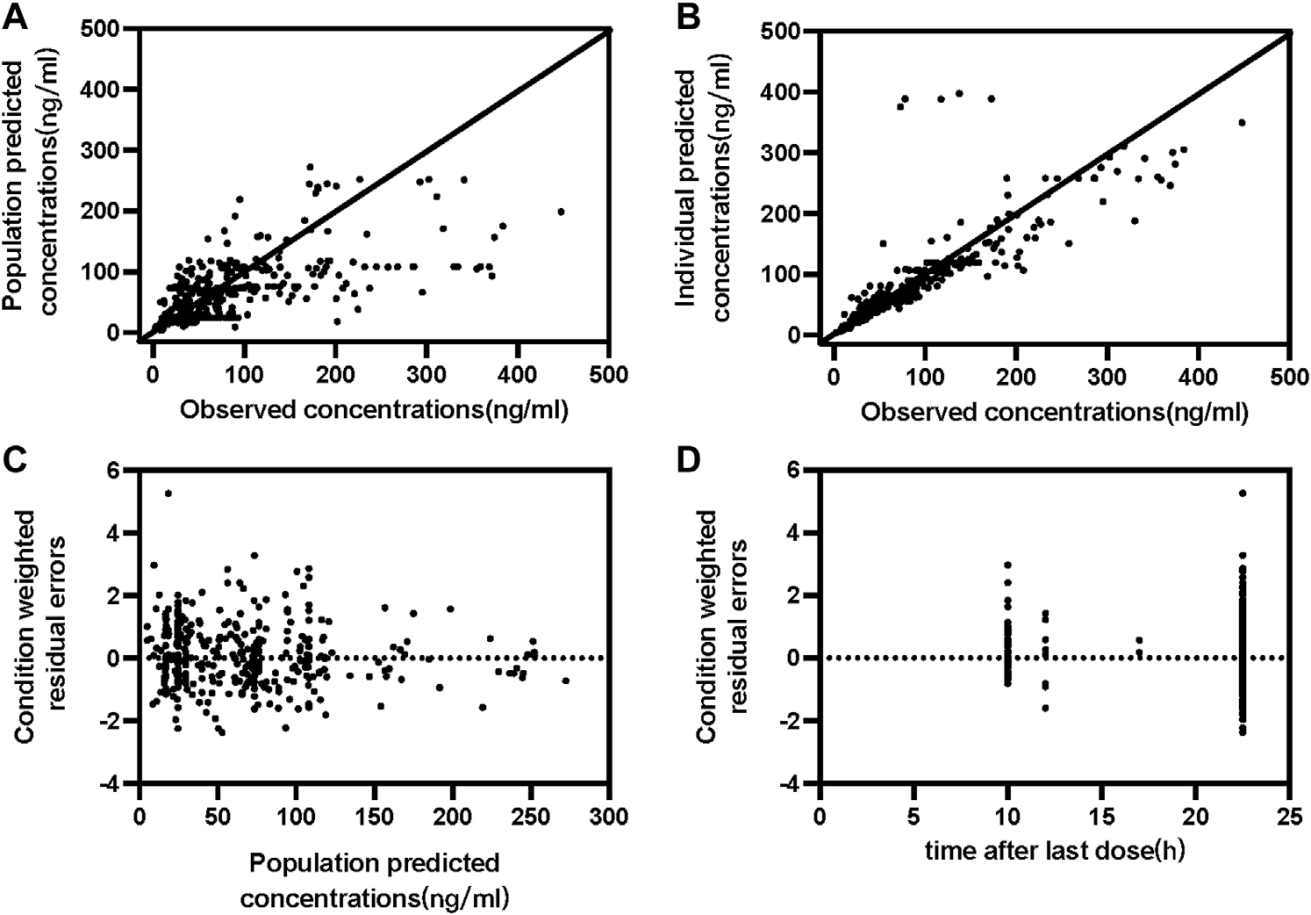
Goodness-of-fit plots of the final population PK model for paroxetine concentration set. **(A)** Observed concentration vs. Population predicted concentrations **(B)** Observed concentration vs. Individual predicted concentrations; **(C)** Conditional weighted residual errors vs. Population predicted concentrations **(D)** Conditional weighted residual errors vs. time after last dose.

One compartment model could fit the PKs characterize of paroxetine. Despite high concentrations tended to be underpredicted and low concentrations tended to be overpredicted, residuals were uniformly distributed around zero (Figure 1). As there were insufficient observations of each subject to estimate PK parameters independently, the Ka was fixed at 0.908/h as previously reported values. The CL/F and V/F in the base model were 21.2 L/h and 8850 L, respectively, as were considered the population typical values. The results of stepwise forward addition and stepwise backward elimination showed that the daily dosage, formulation, and sex showed a statistically significant impact on CL/F and/or V/F. The daily dosage was found to be a major covariate for paroxetine clearance, resulting in a significant decrease in OFV, and the covariate effect of sex about CL/F and V/F also was significant. Furthermore, the formulation had a significant effect on V/F. Since the parameter estimates’ standard errors were under 40% of the estimated value, almost all the parameter estimates could be recognized with reasonable precision. All PPK estimates and bootstrap are summarized in Table 2.

**TABLE 2:** Population pharmacokinetic parameters estimates and bootstrap results of paroxetine.

Parameter	Final model			Bootstrap	
	Estimate	RSE (%)	IIV (CV%)	Median	95% CI
Ka (1/h)	0.908 fixed	-	-	0.908 fixed	-
CL/F (L/h)	21.2	7.2	51.9	21.078	17.63–24.68
V/F (L)	8,850	17.2	83.5	8,226.25	6,180.9–9,924.7
θ_CL-dosage_	-1.03	5.7	-	-0.996	-1.29 - - 0.61
θ_V-formulation_	0.666	9.7	-	0.672	0.53–0.78
θ_F-sex_	0.475	25.3	-	0.440	0.19–0.66
PRO (CV%)	0.0929	-	-	-	-

Ka, first-order absorption rate constant (fixed value); CL/F, apparent clearance; V/F, apparent distribution volume; θ, the factor of the covariate effect; PRO, proportional residual error; RSE, relative standard errors; CI, confidence interval; IIV, interindividual variability.

The following equations can be utilized to define the final PPK model:

$$CL/F\left(L/h\right)=21.2\times {\left(dose/40\right)}^{-1.03}$$

V/F(L)=8850×(1−0.666×formulation)

$$Ka\left(h^{-1}\right)=0.908$$

F1=1+(0.475×SEX)

#### 3.2.2 Model evaluation

**FIGURE 2:**
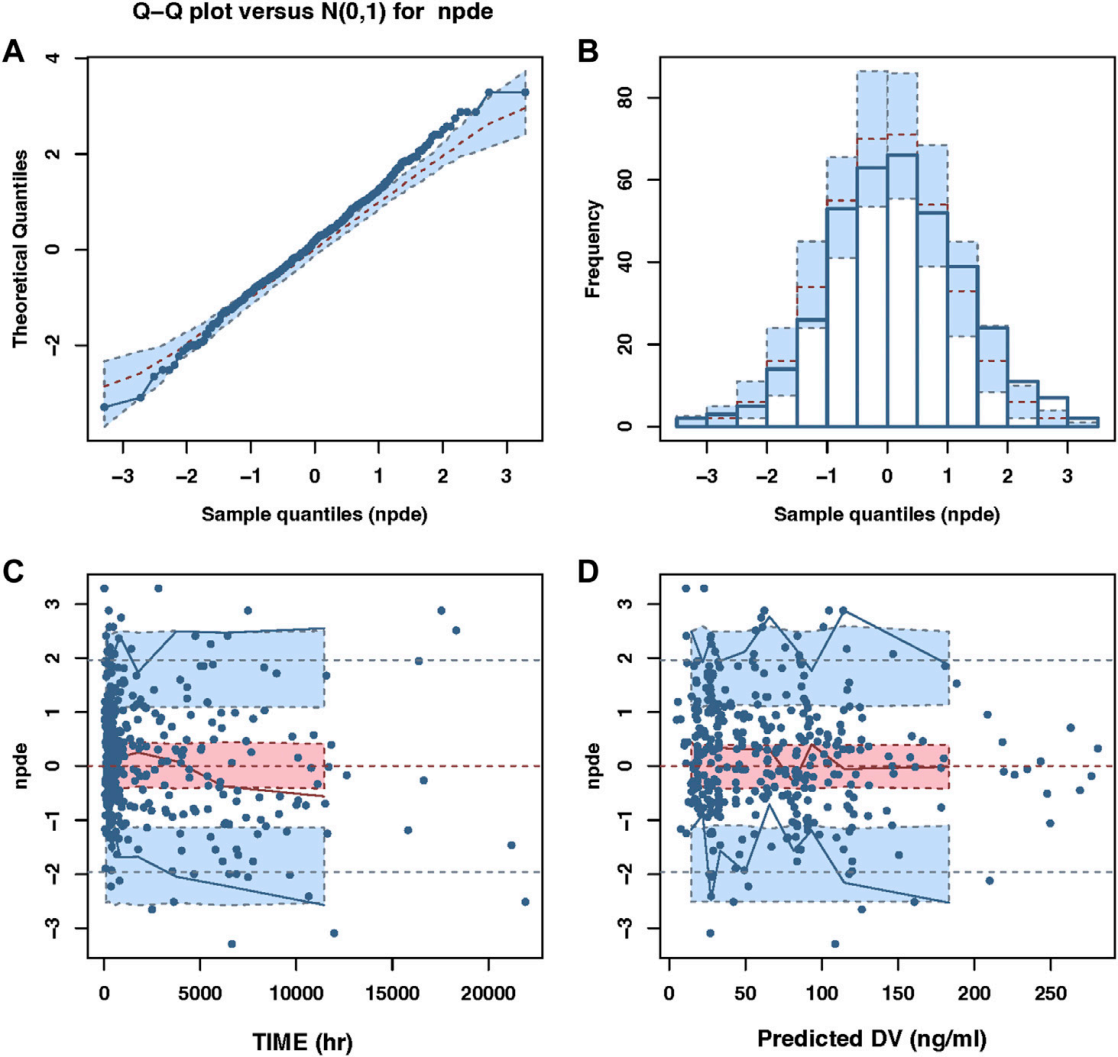
The NPDE plots of the population PK model. **(A)** The quantile–quantile plot **(B)** the distribution histogram of NPDE; **(C)** the NPDE versus time **(D)** the NPDE versus predictions concentration.

The final PPK model’s GOF plots are displayed in Figure 1, and they revealed that the model fit most data satisfactorily when the measured paroxetine concentrations coincided with population- or individual-predicted (PRED) concentrations. The conditional weighted residuals distribution was unbiased in relation to time or population predictions. The NPDE approach was used to assess the paroxetine PK model to validate its predictions. With 1,000 simulations, different daily dose scenarios for each observation were obtained (Figure 2). The quantile-quantile plot (Figure 2A) and the distribution histogram of NPDE (Figure 2B) exhibited a mean and a variance, respectively, of 0.177 and 1.272. Neither NPDE vs. time (Figure 2C) nor NPDE vs. the predicted concentrations (Figure 2D) showed any trend. Furthermore, the final model was relatively stable as seen by the median estimated parameter from the bootstrap program being almost identical to the values from the final model (Table 2). These findings demonstrated that the paroxetine PPK model was generally reliable and precise and that it could be used to predict PPK parameters.

#### 3.2.3 Model simulation

##### 3.2.3.1 Covariates-based simulations for paroxetine concentration

**FIGURE 3:**
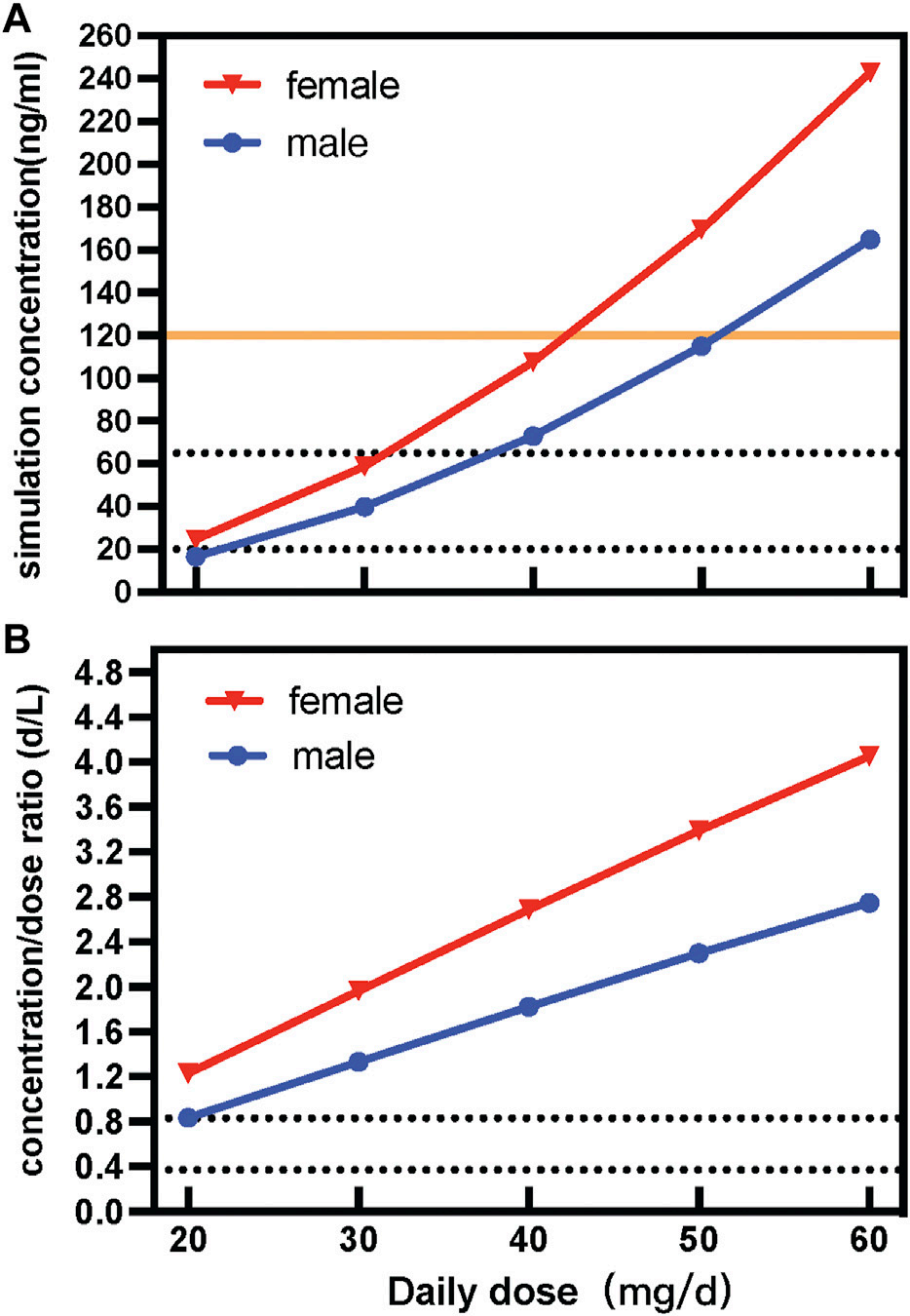
Simulated concentration **(A)** and dose-corrected serum concentrations **(B)** of different daily doses of immediate-release paroxetine tablet.

According to the consensus guidelines for TDM, the reference range for paroxetine is 20–65 mg/L, and the laboratory alert levels is 120 ng/ml. Based on the parameter values of final model, the typical dose courses of steady-state paroxetine concentrations were simulated for males and females after using various daily doses in psychotic patients (Figure 3). The population projected trough concentration could reach 20 ng/ml, the lower limit of the acceptable therapeutic window for paroxetine, with a paroxetine dose of 20 mg QD in the female group, whereas the male group required to be ≥5 mg QD to achieve the same trough concentration. According to the therapeutic reference range (20–65 ng/ml), to maintain better efficacy, the simulation revealed that the dose of immediate-release paroxetine for females was no greater than 30 mg/d and 40 mg/d for males. In addition, the simulation demonstrated that the dose of immediate-release paroxetine at the laboratory alert level (120 ng/ml) should not exceed 50 mg/d for females and 60 mg/d for males (Figure 3A).

Sex variability of immediate-release paroxetine tablets was described by dose-adjusted serum concentrations (C/D ratios; d/L). Contrasting the C/D ratios in male and female patients enabled researchers to investigate the impact of sex. According to the AGNP guideline, the range between the greatest and lowest recorded C/D ratio was used to define Pharmacokinetic variability in patients. The variability of paroxetine was observed to be approximately 1.47-fold in C/D ratios. According to the result of simulation, we observed that with a dose increase, the C/D ratio of a female was higher than that of a male (Figure 3B).

**FIGURE 4:**
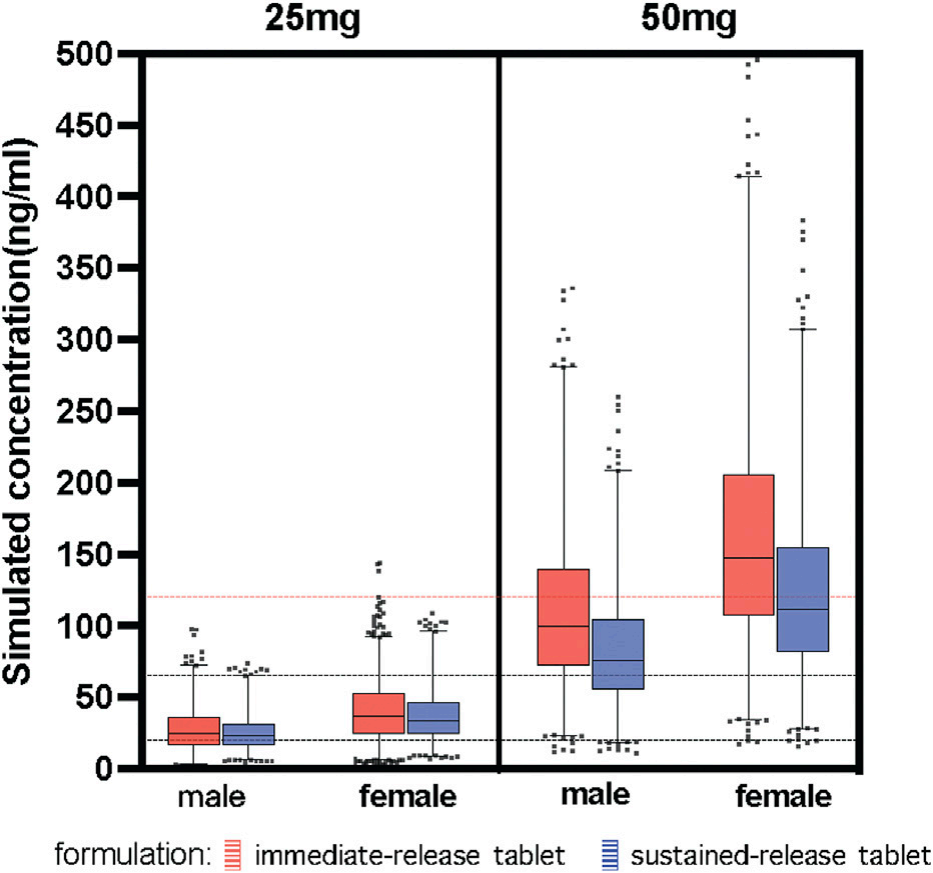
Simulated concentration about covariates identified. Paroxetine concentration stratified by dose (25 mg, 50 mg), formulation (immediate-release tablet, sustained-release tablet) and sex (male, female). The black dash lines represent the upper and lower limit of the therapeutic window (20–65 ng/ml). The red dash line represents the laboratory alert levels (120 ng/ml).

**FIGURE 5:**
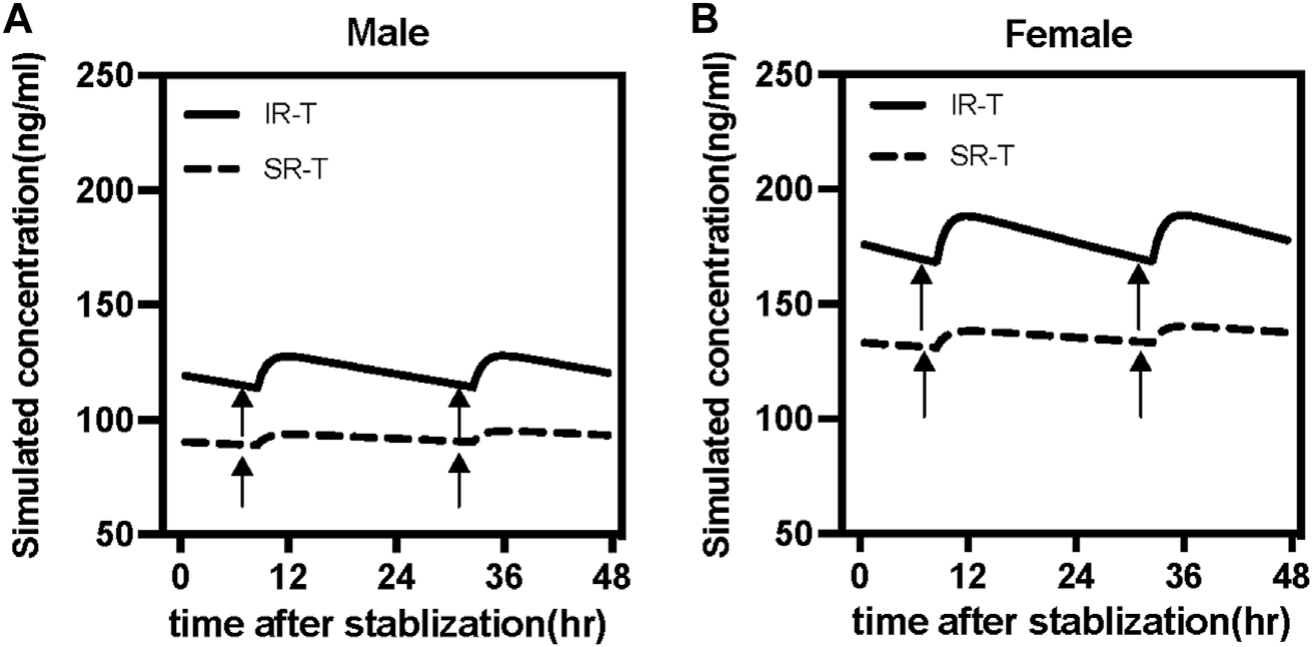
Simulated time courses of paroxetine serum concentration at steady-state under 50 mg daily dose for immediate-release tablet (IR-T, black lines) and sustained-release tablet (SR-T, black dash lines) in males **(A)** and female **(B)**. Black arrows represent the routine sampling time of TDM (7:00 a.m.).

The study simulated different combinations of covariates to assess concentrations under the influence of identified variables (Figure 4). Neither males and females nor different formulations at the same low dosage showed any discernible significant difference. Most paroxetine trough concentrations for different formulations were within the therapeutic window when receiving the dose of 25 mg/d. However, when receiving the high dose of 50 mg/d, trough levels of both immediate- and sustained-release paroxetine were frequently above the therapeutic reference range, and even higher than the alert level. According to the results shown in Figure 4, we observed that the variation at high dose was significant between different formulations. We then simulated the typical time courses of paroxetine concentration of the IR-T and SR-T formulations after receiving 50 mg. The results showed that the trough concentration of IR paroxetine was higher than SR paroxetine, irrespective of female or male (Figure 5).

## 4 Discussion

A one-compartmental model with FOCE-I was developed to describe the PK parameters and predict the concentrations of paroxetine in this analysis. Based on a small number of sample concentrations, we evaluated the effect of demographics and biochemical indicators on the PKs of paroxetine. Finally, based on the final model, we simulated the influence of covariates for different daily doses.

In our study, only one patient received paroxetine at a dosage of 10 mg, meaning that the sample size seemed inadequate to develop a model for the receiving 10 mg paroxetine population. Therefore, we did not initially include patients receiving 10 mg. Lacking data on absorption, with wide individual variation, which resulted in the Ka value being unreliable, we decided to fix the Ka value at 0.908/h. When covariates were filtered with high criteria (*p* < 0.0001), CL/F-dose, V/F-formulation, and bioavailability (F)-sex were determined to be the most important covariates in final model. The theta CL about dosage was a negative value both in the final model and bootstrap, showing that the clearance of paroxetine decreased with an increasing daily dose in this study, similar to earlier studies (Sawamura et al., 2004; Kim et al., 2015). Some previous PPK studies focused on the IR-T, with just one study involving the SR-T, but not including formulation as a covariate (Feng et al., 2006; Findling et al., 2006; Saruwatari et al., 2014; Kim et al., 2015; Nishimura et al., 2016). The understanding of whether antidepressants are susceptible to sex differences has increased along with our understanding of the role that sex differences play in the etiology of depression and PKs (Gibiino and Serretti, 2012). Previous PK analyses showed that sex affected paroxetine clearance or volume of distribution (Feng et al., 2006; Kim et al., 2015; Nishimura et al., 2016); but some studies did not show the differences (Sawamura et al., 2004; Findling et al., 2006; Yasui-Furukori et al., 2011). Age and weight were attempted to be covariates; however, neither had a significant influence on the PKs of paroxetine. Although Jung-Ryul Kim et al. demonstrated that older patients had lower CL/F than younger patients, the median age was 71 years (range: 24–90), which favored older patients (Kim et al., 2015). In our study, the age group was focused on younger patients (median 37.5, range 15–90). Additionally, only six sample patients (3.26%) were older than 71 years, which partially explains the nonsignificant effect of age observed in our study. Although the fact that paroxetine’s safety in adolescents and children has not yet been fully proven, a PK study in this age group indicated that paroxetine was generally safe and well tolerated (Findling et al., 2006; Westergren et al., 2020). Thus, the PPK of paroxetine was mainly focused on patients above 15 years in this study.

When we focused on a combination of the two significant influences of dose and sex, a marked increase in dose-corrected serum concentrations (concentration/daily dose, DRC) was observed. Typically, the dose-corrected values were constant. However, comparing the differences in serum levels of paroxetine between females and males or the concentration/daily dose (C/D) ratio between lower and higher dose patients, higher serum levels in females and high dose patients were observed. The consensus guidelines of the AGNP indicated that DRC factors range from 0.37 to 0.83. However, our study results (DRC: 0.83–4.05) markedly exceeded the reference range, when receiving more than 20 mg of paroxetine, no matter whether IR tablet, or SR tablet was administered. In a PK study of children and adolescents receiving 10–20 mg doses, the concentration in those receiving 20 mg was 6.9-fold higher (2.5–20 ng/ml, DRC: 0.125–1.02) than the concentration in those receiving 10 mg (Findling et al., 1999). Another study of healthy subjects combined 20 mg paroxetine with Fosamprenavir-Ritonavir, and the C_min_ was 0.017–0.03 mg/L (DRC: 0.85–1.5) (van der Lee et al., 2007). Our results were close to those in the above report in individuals receiving 20 mg, where the DRC ranged from 0.83 to 1.23. Furthermore, the steady concentration changed 25-fold (25–670 nmol/L, DRC: 0.262–7.023) in some fast extensive metabolizers and elderly poor metabolizers receiving 30 mg, and the concentration/dose ratios increased throughout the dose levels from 10 to 50 mg once daily, which was also similar to our findings (Sindrup et al., 1992). It could be considered that guideline reference ranges are appropriate at low doses, while individual differences are more obvious at high doses. Given that its initial definition was based on limited evidence that might not accurately characterize the relationship of concentration-response in patients receiving high dose paroxetine, the reference value is a contentious concept. There is no doubt that the reference ranges given for most psychotic patients are from guidelines (Hiemke et al., 2018). Some patients would benefit from concentration above the upper limit or below the lower limit of the AGNP recommended ranges. However, for most patients receiving paroxetine in our study, reference suggestions were lacking.

In addition, covariate analysis discovered that the male relative bioavailability was lower than that of female subjects. This is consistent with a prior study that reported that female patients had a higher median serum paroxetine level than male patients (Feng et al., 2006; Nishimura et al., 2016). Bioequivalence analyses revealed sex differences in relative bioavailability (Lu et al., 2019). This suggests that females need a lower paroxetine dose than males, indicating that females receiving a paroxetine dose of 20 mg/d should be 25 mg/d in males to achieve the same lower limit of the therapeutic reference range. Moreover, there is a trend here that as the dose increases, the difference between genders is pronounced, resulting in the C/D ratio of females being 1.47-fold higher than males.

One of the distinguishing features of paroxetine is nonlinear dynamics, and this may be related to a decline in inherent clearance caused by saturation of the high-affinity enzyme *CYP2D6*, as well as time-dependent metabolism caused by auto-inactivation of the enzyme (Sindrup et al., 1992; Feng et al., 2006; Findling et al., 2006; Kim et al., 2015). *CYP2D6* is inactivated by metabolic or mechanism-based inhibition (Jornil et al., 2010; Mikami et al., 2013; Uttamsingh et al., 2015). A scaling model for mechanism-based inactivation by paroxetine from *in vitro* data suggested that patients receiving paroxetine at doses of 20–30 mg QD should experience a 93% inactivation of *CYP2D6* (Venkatakrishnan and Obach, 2005). Additionally, as previously reported, it is also recognized that *CYP2D6* genetic polymorphism is a major factor affecting the paroxetine PK variability (Feng et al., 2006; Kim et al., 2015; Nishimura et al., 2016; Chen et al., 2017). Therefore, we evaluated the effect of *CYP2D6* genotypes on the PK parameters. Due to the limitation in sample sizes, when the patients were classified by genotype, we were unable to discover any appreciable influence of *CYP2D6* polymorphism on the correlation between plasma levels and the daily dose. A previous study showed that the differences in plasma paroxetine concentrations resulting from different *CYP2D6* genotypes were manifested at lower doses (10 mg/day) in the Japanese population but were not observed at higher doses (Sawamura et al., 2004). The same conclusion was drawn in another study that showed that genetic polymorphism was related to PKs at lower doses (Saruwatari et al., 2014). According to previous PK models, the paroxetine’s mechanism-based suppression of *CYP2D6* could account for the nonlinear and accumulation properties of paroxetine (Venkatakrishnan and Obach, 2005; Johnson et al., 2009; Mikami et al., 2013). *CYP2D6* inhibition was associated with higher doses of paroxetine (such as 30 mg) (Sindrup et al., 1992; Carvalho Henriques et al., 2020), and our study dose groups were focused on 20 mg or 40 mg. Furthermore, due to PK overlap amongst phenotypic groups, genetic polymorphism did not accurately reflect the change in PKs over time or the effects of drug-drug interaction. Genetically extensive metabolizers may not represent a homogeneous group, and clinical practice should take available genetic data into account (Storelli et al., 2018). Previous studies revealed that extensive metabolizers have the potential to convert to poor metabolizers during paroxetine treatment (Jeppesen et al., 1996; Brosen, 2015). A study found that a higher rate of phenotypic conversion is especially observed in heterozygotes than in homozygotes (94 vs. 56%) (Storelli et al., 2018). Thus, self-inhibition and potential phenotypic transformation of *CYP2D6* may lead to nonlinear kinetics of certain genotypes at common doses (Hicks et al., 2015). Consequently, the role of paroxetine as a *CYP2D6* inhibitor would rise as the paroxetine dose was increased and caused a decline in enzyme activity (Findling et al., 2006). This potentially partly explains the role of genotypes being nonsignificant at high paroxetine doses. Further study is needed with more patients to evaluate the effect of genotype in the Chinese population, combined with clinical efficacy and adverse effects. Close clinical monitoring is preferred supported by serum concentration measurements (Westin et al., 2017). It is more accurate to measure serum concentration where *CYP2D6* genetic polymorphism was not identified. Combining it with the genotyping could make the consequence more credible, if easily accessible (Westin et al., 2017), and may be useful in obtaining more exact PK parameters as well as determining whether anomalous serum concentrations were caused by noncompliance.

In the final model, most PK parameters had moderate interpatient variability, and mainly the apparent distribution volume exhibited significant formulation variability. There are two approved formulations of paroxetine: 20 mg per tablet and 25 mg per tablet (enteric-coated sustained-release tablets). There are no significant differences in clearance across various formulations of the same drug. But inter-patient variability in paroxetine trough levels was different for the tablet and enteric-coated sustained-release tablets. Typically, the absorption rate constant of the paroxetine SR-T was equivalent to that of paroxetine IR-T due to the extended-release features of the sustained-release formulation, however, its relative bioavailability was 0.67 depending on the product brochures of paroxetine (GlaxoSmithKline, 2005). This means that different formulations have an effect on the volume distribution. Following absorption, the typical value of the apparent V/F was 8850 L, indicating that paroxetine is extensively distributed in the body. Compared with the IR-T, the SR-T resulted in a decrease in V/F of about 66.6% in this study. The V/F differed, which meant a higher serum concentration for paroxetine IR-T than SR-T. Our study found that the difference was nonsignificant between IR-T and SR-T at lower doses (such as 25 mg), no matter whether females or males; but, when receiving a higher dose (such as 50 mg), the difference was obvious. Notably, if female patients received more than a 40 mg dose, they easily exceeded the laboratory alert level; thus, another formulation such as SR-T could be considered. Furthermore, when females receive a high-dose paroxetine IR-T, there is a clinical need to monitor the dynamic change in serum concentration and make prompt adjustments.

Dose, sex, and formulation have a significant influence on the serum concentrations of paroxetine, and additive effects must be considered. Thus, we estimated the effects of these covariates in our study, which were dose-related (2.18-fold), formulation-related (1.28-fold), and gender-related (1.7-fold), respectively. Furthermore, rather than a set reference range to evaluate the impact of paroxetine on disease, we recommend using an individual therapeutic range considering the non-linear concentration fluctuations caused by dose increases. The tendency in antipsychotic treatment is to shift away from reference ranges to individual therapeutic ranges, which may help produce an optimal response in the patient.

Some limitations should be addressed in more research. During the concomitant drug screening, this study did not identify potential agents that significantly affected the plasma concentration of paroxetine. However, it is worth noting that, as a strong inhibitor of the CYP2D6 enzyme, paroxetine may have a strong inhibitory effect on drugs also metabolized by the CYP2D6 enzyme. In addition, the high protein binding rate of paroxetine may potentially increase the free concentration of paroxetine when administered with another drug with a higher binding rate of plasma proteins. Moreover, some classifications could not be fully analyzed because we only retrospectively got a small sample of patients’ TDM concentrations for paroxetine. Furthermore, because of the prevalence of selection bias in retrospective data gathering, it was possible to include some subjects whose paroxetine treatment was ineffective, meaning that they were tolerated despite receiving higher daily doses. Some patients’ information was not included; thus, the results of some influencing factors were not reflected, such as genotype. In summary, these factors may, in part, contribute to the bias in estimates.

## 5 Conclusion

This sparse data paroxetine PPK model describes the observed paroxetine PK data in Chinese psychotic patients. The model characteristically describes the non-linear increase in paroxetine concentration. The PKs of paroxetine were affected by dose, formulation, and sex among the variables assessed. PPK models may be useful in developing individual treatment regimens for patients, as well as offering insight into variables influencing patient medication variability. Under the impact of covariates, the risk of clinical concentrations over the therapeutic level was dramatically enhanced. Trough concentrations should be monitored, and dosage adjusted to keep individual patient exposure to be safe.

## Data Availability

The raw data supporting the conclusions of this manuscript will be made available by the authors, without undue reservation.
